# Effect of Fucoidan on Structure and Bioactivity of Chinese Steamed Bread

**DOI:** 10.3390/foods13071057

**Published:** 2024-03-29

**Authors:** Qingyu Yang, Man Li, Chenqi Gu, Anni Lu, Lijun Dong, Xiling Zhang, Xiufa Hu, Yao Liu, Jun Lu

**Affiliations:** 1 College of Grain Science and Technology, Shenyang Normal University, Shenyang 110034, China; yangqy0311@163.com (Q.Y.);; 2 State Key Laboratory of Food Nutrition and Safety, Shenyang Normal University, Shenyang 110034, China; 3 Pinehurst School, Albany, Auckland 302-308, New Zealand; 4 Beijing Imperial Food Garden Food Co., Ltd., Beijing 101407, China; 5 Auckland Bioengineering Institute, University of Auckland, Private Bag 92019, Auckland 1142, New Zealand; 6 Department of Food and Agriculture Technology, Yangtze Delta Region Institute of Tsinghua University, Jiaxing 314006, China

**Keywords:** fucoidan, *Undaria pinnatifida*, Chinese steamed bread, starch digestibility, antioxidant activity

## Abstract

Fucoidan refers to a group of sulphated polysaccharides obtained from brown seaweed, with numerous biological activities. In this study, fucoidan was fortified into Chinese steamed bread (CSB) at different concentrations (0, 1%, 3% and 5%) and the effect of fucoidan on the dough properties, structure properties and bioactivity were investigated. The results showed that fucoidan could change the viscosity of unfermented dough, and a high concentration of fucoidan could remove the free radicals produced by the SH–SS exchange reaction (GS-) in the dough, which significantly reduced the content of disulfide bond and reduced the expanded volume of fermented dough (*p* < 0.05). In addition, fucoidan forms a physical barrier on the surface of starch particles and hinders the reaction between protein-to-protein; therefore, fucoidan increased the hardness, gumminess and chewiness in CSB, and reduced the specific volume in CSB. Furthermore, the fucoidan-fortified CSB samples were found to have both the ability to significantly reduce the predicted glycemic index (pGI) (*p* < 0.05) and improve antioxidant activity (*p* < 0.05). Collectively, these findings could provide a theoretical basis for the applications of fucoidan as a functional component in fermented foods.

## 1. Introduction

Chinese steamed bread (CSB) is a major staple food in China [1]. The basic ingredients of CSB are flour, yeast and water, and it has unique eating and nutritional qualities [2]. As compared with most traditional breads, CSB has a low oil and salt content and a low ripening temperature; therefore, CSB can maintain its unique nutritional characteristics and sensory quality [3]. Depending on the area and consumer preferences, CSB might have different textures such as dense, extremely solid, fluffy and soft. In general, CSB in northern China, such as Shandong and Henan, is firm and chewy, whereas CSB in southern China, such as Hubei Province, is softer and has a more open structure [1]. However, the CSB is a high glycemic index (GI) food [4]. In China, around 21 million tons of wheat flour is used to make CSB, which accounts for 30% of the country’s total wheat flour production [5]. Thus, CSB has a wide consumer base and high market demand in China. Studies have found that consumption of high-GI foods might lead to a rapid rise in blood sugar levels and a rapid increase in insulin secretion, which would not be beneficial to people with diabetes [6]. At present, the incidence of type 2 diabetes is increasing in the youth population, and the total number of diabetes patients in China is estimated to reach 110 million [7,8]. Accordingly, the development of CSB with nutritious and healthy qualities may be a promising way for people to meet their glycemic control needs.

Fucoidan is a sulfated polysaccharide isolated mainly from brown algae, with α(1→3) and α(1→4) linked a-l-fucopyranose backbone and has attracted attention for its outstanding excellent biological activity [9,10]. Previous studies results have considered that fucoidan had anti-diabetic effects and could prevent complications related to diabetes mellitus [11]. Furthermore, fucoidan from different seaweeds also showed different inhibitory effects on starch hydrolases (e.g., α-amylase, α-glucosidase and amyloglucosidase) [12]. Recent studies have found that fucoidan alters the structure of starch, increasing its viscosity and intermolecular interactions, thereby inhibiting the release of glucose during digestion [13]. In addition, fucoidan also has antioxidant properties [14]. Koh et al. [15] found that low molecular weight fucoidan has stronger antioxidant properties when compared to other components. However, most of the current studies on fucoidan have focused on its structure or activity, but less on its application in fermented foods.

Polysaccharides could influence the internal protein and starch structure of flour products. Earlier research has shown that hydrocolloid molecules interact with starch to regulate the overall quality of the flour product by controlling moisture and limiting water mobility, which provides the proper texture to the final product [16]. Recent research has indicated that the high water-holding capacity of wheat bran dietary fiber (WBDF) is beneficial for the formation of the gluten network structure, improving the elasticity and extensibility of the flour product. Moreover, an appropriate amount of WBDF might interact with gluten proteins to form complex gluten structures [17]. Kong et al. [18] found that non-starch polysaccharides increase or decrease the peak viscosity to inhibit granule swelling and leaching of amylose, which inhibit the disintegration of starch granules and reduce the digestibility of starch. The action of soluble polysaccharides might cover the surface of amylopectin or entangle with amylopectin molecules, thus inhibiting starch crystallization in flour products [19]. Furthermore, polysaccharides could also improve the texture, viscosity and sensory properties of flour products [20] and increase their antioxidant property. Hamdani et al. [21] found that the DPPH· radical scavenging capacity of the flour product formulation increased from 10.61 to 16.83% with the addition of polysaccharides. Therefore, it is very meaningful to study the effect of adding fucoidan on the structure and biological activity of CSB.

Currently, there is limited theoretical research on the influence of fucoidan on the structure of wheat food products. In this research, we study the potential of different amounts of fucoidan (0, 1%, 3% and 5%) as a functional ingredient of CSB by characterizing the effect of the dough properties, structure properties and functional properties of CSB. The aim of this study was to investigate the interplay among fucoidan, gluten proteins and starch in order to comprehend the underlying mechanisms that impact the dough properties, structure properties and functional properties of CSB. The findings of this study could provide a theoretical basis for the applications of fucoidan in fermented foods.

## 2. Materials and Methods

### 2.1. Materials

Wheat flour and yeast were purchased from Biyoute market (Shenyang, China). Fucoidan isolated from the brown seaweed species, Undaria pinnatifida, was purchased from Auckland, New Zealand. Glycine was purchased from Saiguo Biotech Co., Ltd. (Guangzhou, China) and trichloroacetic acid was obtained from Eon Chemical Technology Co., Ltd. (Shanghai, China). Artificial saliva, artificial gastric fluid (pH 1.5) and artificial intestinal fluid (pH 6.8) were purchased from Chuangfeng Automation Technology Co., Ltd. (Dongguan, China). 3,5-Dinitrosalicylic acid (DNS) reagent was purchased from Coolaber Science & Technology Co, Ltd. (Beijing, China). 1-Diphenyl-2-picrylhydrazyl (DPPH) was obtained from Ruiyong Biotechnology Co., Ltd. (Shanghai, China). The starch content assay kit was obtained from Solarbio Science & Technology Co., Ltd. (Beijing, China). 2,3-Dihydroxysuccinic acid and β-mercaptoethanol were purchased from Macklin Biochemical Co., Ltd. (Shanghai, China). Urea, guanidine hydrochloride (GuHCl), 5,5′-dithiobis-2-nitrobenoic acid (DTNB), α-amylase (5 U/mg), pepsin (30,000 U/g), pancreatin (4000 U/g), amyloglucosidase (100,000 U/mL) and 2,2′-azinobis-(3-ethylbenzthiazoline-6-sulphonate) (ABTS) were obtained from Yuanye Bio-Technology Co., Ltd. (Shanghai, China). 

### 2.2. Preparation of Dough

To prepare the dough, 200 g of wheat flour, different qualities of fucoidan (0, 1%, 3%, and 5% based on wheat flour) and 3 g of yeast were dissolved into 100 mL of water and stirred in a mixer to make fermented dough, named FWD-0, FWD-1, FWD-2 and FWD-3, respectively. In contrast, to prepare the unfermented dough, we only dissolved wheat flour and fucoidan into water and stirred, named UWD-0, UWD-1, UWD-2 and UWD-3, respectively. 

### 2.3. Preparation of Chinese Steamed Bread (CSB) 

To prepare the CSB, 200 g of wheat flour, different qualities of fucoidan and 3 g of yeast were dissolved into 100 mL of water and stirred in a mixer to make fermented dough. The dough was fermented at 38 °C and 75% relative humidity for 30 min. The dough was divided into 50 g and fermented in a fermentation cabinet at 38 °C and 75% humidity for 15 min. Finally, the samples were steamed for 15 min. Then, the samples were cooled for 1 h at room temperature for further analysis. The additions of 0, 1%, 3% and 5% fucoidan to CSB were named CSB-0, CSB-1, CSB-2, and CSB-3, respectively. Part of the CSB samples were dried at 45 °C overnight and grounded into powder to pass through a 150 μm sieve. Other CSB samples were used for textural property analysis, SEM analysis and sensory analysis.

### 2.4. Dough Properties

#### 2.4.1. Rheological Properties

The rheological properties’ patterns of the unfermented dough samples were determined using a Rheofermentometer (DHR-1, TA Corporation, New Castle, DE, USA) according to the method of Cao et al. [22] with slight modification. The plate and plate system were used with a diameter of 40 mm and a gap of 1 mm between the plates. A strain of 0.1% was applied during the test. The test temperature was 25 ± 1 °C, and the test frequency was in the range of 0.1–40 Hz. 

#### 2.4.2. Determination of Free Sulfhydryl (SH) and Disulfide Bond (SS) Content

The content of free sulfhydryl group and disulfide bond in the fermented dough samples was determined following the method outlined by Beveridge et al. [23] with slight modification. First, 150 mg of dough samples were suspended in 1.0 mL of tris-glycine buffer at pH 8.0. Then, 4.7 g of guanidine hydrochloride was added to the mixture and diluted to 10 mL with the buffer. Next, 1 mL of sample dispersion was combined with 4 mL of urea-guanidine hydrochloride (8 mol/L urea + 5 mol/L guanidine hydrochloride) and 0.1 mL of 4 mg/mL 5,5′-dithiobis-2-nitrobenzoic acid (DTNB), and the absorbance of reaction mixture was measured at 412 nm to calculate the free SH content. Additionally, 1 mL of sample dispersion, 0.1 mL of β-mercaptoethanol and 4 mL of urea-guanidine hydrochloride was mixed and incubated in a dark environment for 1 h. Then, 10 mL of 12% trichloroacetic acid (TCA) was introduced to the mixture and incubated for 4 h. The samples were subjected to centrifugation at 8000 r/min for 10 min at room temperature. The sediment was washed with 12% TCA (5 mL) and centrifuged two times at 8000 r/min for 10 min. Following this, the precipitate was dissolved in 10 mL of 8 mol/L urea solution and 0.08 mL of 4 mg/mL DTNB was added. The absorbance of the sample was measured at 412 nm to calculate the total SH content. Then, the SS was calculated according to the following formula:

(1) $${\mathrm{SH}}_{\text{total/free}}\;(\mu mol/g)=\frac{\left(73.53\times A_{412}\times D\right)}{C}$$

(2) $$\mathrm{SS}\;(\mu mol/g)=\frac{{\mathrm{SH}}_{\mathrm{total}}-{\mathrm{SH}}_{\mathrm{free}}}{2}$$

A_412_ was the absorbance of protein fractions; C was the concentration of the sample; D was the dilution factor of the sample. In addition, D was 5.02 and 10 for the free SH and total SH contents, respectively.

#### 2.4.3. Determination of Expansion Volume of Dough

First, 80 g of dough was placed a 250 mL graduated cylinder. Subsequently, the dough was fermented in a fermentation cabinet at 38 °C and 75% humidity for 45 min. The fermentation volume of the dough in the graduated cylinder was recorded. The expansion volume of dough was represented by the difference between the final volume of the dough after fermentation and the initial volume of the dough [24].

### 2.5. Textural Property Analysis (TPA)

The textural properties of the CSB were measured using a CT3 texture analyzer (CT3-4500, Brookfield, WI, USA) with a flat surface P/36R cylinder probe. Test parameters: trigger force: 5.0 g; sample deformation: 20%; test-speed: 1.0 mm/s; number of compressions: two cycle compression [25]. The measurements were conducted at least three times.

### 2.6. Specific Volume of Chinese Steamed Bread (CSB) 

After steaming and cooling, the CSB was first placed in a beaker (known volume, V_C_). The container was then topped with millet and the CSB was removed to record the volume of millet, V_B_. The weight of the CSB was recorded as W_D_. The specific volume of CSB (Vs) was the following formula [26]:

(3) $$V_{S}\;({\mathrm{cm}}^{3}/g)=\frac{V_{C}-V_{B}}{W_{D}}$$

### 2.7. Fourier Transform Infrared (FT-IR) Spectroscopy

First, 200 mg of CSB was uniformly spread on the surface of diamond attenuated total reflection (ATR) crystal The CSB were scanned by using a FT-IR spectrometer (Tensor II, Bruker, Karlsruhe, Germany) at a resolution of 4 cm^−1^ in the 400 cm^−1^ to 4000 cm^−1^. In addition, the data points were analyzed using the PeakFit v4.12 software for the amide III region between 1330–1220 cm^−1^ [10,27].

### 2.8. X-ray Diffraction (XRD)

The crystallization structure of CSB-0, CSB-1, CSB-2 and CSB-3 was determined using an X-ray diffractometer (Ultima IV, Rigaku Japan Science, Tokyo, Japan). The samples were scanned from 4° to 45° in step angles of 4 °/min at 40 kV and 30 mA Cu-Ka radiation. Furthermore, the relative crystallinity of the samples was analyzed using the Jade 6.0 software [28].

### 2.9. Scanning Electron Microscope (SEM)

The scanning electron microscope (S-4800, Hitachi, Tokyo, Japan) was used to analyze the microstructural morphology of the CSB samples. The dried samples were deposited on a copper stub using double-sided adhesive tape, which was then sprayed with gold under a vacuum. SEM images of the CSB samples were taken at 30× and 200× magnifications with an accelerating voltage of 1.0 kV.

### 2.10. In Vitro Starch Digestibility of CSB

In vitro starch digestibility was determined according to the method described by Gao et al. [29] and Koh et al. [30] with some modifications. Specifically, 4 g of different samples (CSB-0, CSB-1, CSB-2, CSB-3) were mixed with 4 mL of artificial saliva (pH 7.0) containing α-amylase (75 U/mL of final enzymatic activity) in a 37 °C water bath. The mixture was stirred using a magnetic stirring bar for 2 min. Subsequently, the mixture was mixed with 8 mL of artificial gastric fluid (pH 3.0) containing pepsin (2000 U/mL of final enzymatic activity) and incubated for 2 h. Following this step, the mixture was mixed with 16 mL of artificial intestinal fluid containing amyloglucosidase (21 U/mL of final enzymatic activity) and trypsin (100 U/mL of final enzymatic activity) and stirred for 5 s. The pH of the mixture was adjusted to 7.0, transferred into a dialysis tube with a cut-off molecule of 14 kDa and placed in 200 mL of PBS buffer. The mixture was incubated at 37 °C and 200 rpm for 3 h.

Furthermore, 0.5 mL of dialysate (supplemented PBS) was collected at 0, 5, 10, 15, 30, 45, 60, 75, 90, 120, 150, 180 min throughout the intestinal period. Then, 0.5 mL of dialysate was mixed with 0.5 mL of DNS and the mixture was boiled in boiling water for 5 min. The mixture was cooled at room temperature and supplemented with 4 mL of water in a test tube. Following this step, the absorbance of each sample was assessed at 540 nm using a UV-1200S spectrophotometer. Wheat white bread was used as a reference (HI = 100). The calculation of the predicted glycemic index (pGI) and hydrolysis index (HI) was performed according to the following:

(4) $$\mathrm{HI}=\left(\frac{{\mathrm{AUC}}_{\mathrm{sample}}}{{\mathrm{AUC}}_{\mathrm{wheat}\;\mathrm{bread}}}\right)\times 100$$

pGI = 0.549 × HI + 39.71(5)

AUC was the area under the starch hydrolysis rate curve of the samples.

### 2.11. Total Starch Test

The total starch of the CSB was measured using a starch content assay kit (Solarbio, Beijing, China). The concentrations were detected according to the manufacturer’s instructions. In addition, the results were reported as optical density (OD) with a UV-1200S spectrophotometer.

### 2.12. Kinetics of Starch Hydrolysis

Gao et al. [29] found that the digestion curve generated by the in vitro digestion system conformed to a first-order reaction. The rate of starch digestion was quantified as the percentage of total starch hydrolyzed at various time points of intestinal digestion. The starch digestion curve was modeled using a first-order rate equation proposed by:

(6) $$C=C_{\infty }\left(1-e^{-\mathrm{kt}}\right)$$

C was the percentage of digested starch at time; C_∞_ was equilibrium percentage of starch digestibility at the end of the digestion; k was the first-order rate constant of the percentage of digested starch; t was the time.

### 2.13. Fluorescence Quenching

First, 0.5 g of CSB were mixed with 25 mL of PBS buffer and 5 mL of α-amylase or amyloglucosidase. The mixture was incubated for 30 min at 37 °C. Then, the samples were centrifuged at 8000 r/min for 5 min and the supernatant was collected for testing. The excitation wavelength of the supernatant was set at 288 nm, and the emission signals were recorded from 310 to 400 nm with the widths of 10 nm for both excitation and emission slits [31]. 

### 2.14. Antioxidant Activity

#### 2.14.1. DPPH· Radical Scavenging Activity

Briefly, 0.5 g of CSB sample was dissolved in 15 mL of distilled water. Then, 1.4 mL of ethanol 0.1 mM DPPH· solution was mixed with 0.1 mL of CSB solution. These solutions were vortexed for 1 min and kept for 30 min in a dark environment at room temperature. The supernatant was collected for testing after centrifugation at 8000 r/min for 5 min. The absorbances were estimated using a UV-1200S spectrophotometer at 517 nm. Then, the percentage scavenging of DPPH· was determined according to the following formula [32]:

(7) $$\mathrm{Percentage}\;\mathrm{scavenging}\;\mathrm{of}\;\text{DPPH·}=\left[1-\frac{\left(A_{1}-A_{2}\right)}{A_{0}}\right]\times 100$$

A_0_ was the absorbance of the DPPH· solution without the sample; A_1_ denotes the absorbance for sample with DPPH·; A_2_ was the absorbance for sample without DPPH·.

#### 2.14.2. ABTS· Radical Scavenging Activity

The ABTS· solution was diluted with ethanol to a final absorbance of the control of 0.70 ± 0.01 at 734 nm before use. Briefly, 0.5 g of CSB sample was dissolved in 15 mL of distilled water. Subsequently, 1.4 mL of the ABTS· solution was combined with 0.1 mL of the CSB solution. These solutions were vortexed for 1 min and kept for 5 min in a dark environment at room temperature. Then, the samples were centrifuged at 8000 r/min for 5 min and the supernatant was collected for the test. The absorbances were estimated using a UV-1200S spectrophotometer at 734 nm. The percentage scavenging of ABTS· was determined according to the following formula [32]:

(8) $$\mathrm{Percentage}\;\mathrm{scavenging}\;\mathrm{of}\;\text{ABTS·}=\left[1-\frac{\left(A_{1}-A_{2}\right)}{A_{0}}\right]\times 100$$

A_0_ was the absorbance measured for the ABTS· solution without the sample; A_1_ denotes the absorbance for sample with ABTS·; A_2_ was the absorbance for sample without ABTS·.

### 2.15. Sensory Analysis

#### 2.15.1. Electronic Tongue Analysis

Electronic tongue analysis was performed using a Taste Sensing System SA 402B (Intelligent Sensor Technology, Inc., Kanagawa, Japan). First, 100 g of the CSB sample was homogenized in 300 mL of distilled water for 10 min. Subsequently, the mixture was centrifuged at 8000 r/min for 5 min, and the supernatant was collected [33].

#### 2.15.2. Descriptive Sensory Analysis

The training and methods of descriptive sensory analysis were conducted with reference to previous studies [34,35,36]. After steaming, the samples were cooled for 1 h at room temperature. Then, 10 trained volunteers were invited to assess and score the CSB samples for color, porosity, odor, texture and flavor richness. The samples were presented individually to the volunteers using cardboard. It should be noted that the volunteers rinsed their mouths with plain water after testing each sample. The descriptive sensory analysis table was made according to references [34,35], as shown in Table 1.

### 2.16. Statistical Analysis

All data are presented as mean ± SD, and all analyses and graphsare created using Origin 2022 (OriginLab Corp., Northampton, MA, USA) and the SPSS 27 (IBM Corporation, New York, NY, USA). One-way analysis of variance (ANOVA) followed by Duncan’s multiple range test was performed for all the statistical analyses, and *p* < 0.05 was considered statistically significant. 

## 3. Results and Discussion

### 3.1. Effect of Fucoidan on the Rheological Properties of Dough

**Figure 1:**
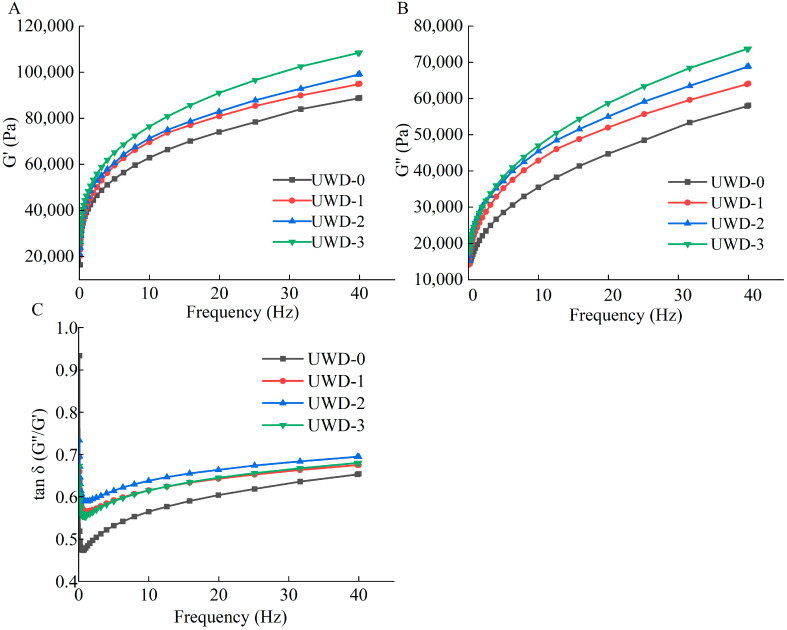
Effects of fucoidan on the rheological properties of dough. (**A**) G′, storage modulus, (**B**) G″, loss modulus, and (**C**) tan δ, loss tangent. UWD-0, unfermented wheat dough without fucoidan; UWD-1, unfermented wheat dough with 1% fucoidan; UWD-2, unfermented wheat dough with 3% fucoidan; UWD-3, unfermented wheat dough with 5% fucoidan.

Figure 1 illustrates the impact of fucoidan on the rheological properties of dough. As shown in Figure 1A,B, the values of G′ and G″ increased with the increase in frequency in all samples within the 0.1–40 Hz range. The elasticity of the dough was denoted by the storage modulus (G′), indicating the extent of deformation, while the loss modulus (G″) reflected the viscosity properties [37]. The value of G′ was higher than the value of G″ throughout the frequency range for UWD-0, UWD-1, UWD-2 and UWD-3, indicating the predominant solid-like behaviors within the dough [38]. Tan δ is obtained by G″/G′, which indicates the relationship between G′ and G″. The higher the value of tan δ in the dough, the higher the viscosity prevailed in the doughs and the higher the gluten protein network weakened [39,40]. As shown in Figure 1C, the values of tan δ ranged between 0.1 and 1 in UWD-0, UWD-1, UWD-2 and UWD-3. UWD-1, UWD-2 and UWD-3 had higher values of tan δ compared with those in UWD-0. The above results showed that the viscosity of the dough increased and the gluten network weakened as fucoidan added.

The increase in the viscosity of the dough was due to the weakening of the gluten protein. The water competition between polysaccharide and gluten protein, as well as the inhibition of the formation of interchain and intrachain disulfide bonds in the gluten chain, were the main reasons for the weakening of the glutenin network [41,42,43,44]. Therefore, fucoidan might inhibit the formation of the protein network by competing with gluten protein for water and hindering the formation of gluten protein disulfide bonds, thus enhancing the viscosity of the dough.

### 3.2. Analysis of Free Sulfhydryl (SH), Disulfide Bond (SS) Content and Expansion Volume

The content of free sulfhydryl and disulfide bond was an important indicator to judge the strength of the gluten network [10]. The higher content of disulfide bond could form a solid gluten network structure [45]. As shown in Table 1, the content of free sulfhydryl was significantly increased in FWD-1, FWD-2 and FWD-3 when compared with FWD-0 (*p* < 0.05). With the addition of fucoidan, the free sulfhydryl contents of the dough were increased by 2.90%, 8.70% and 15.46%, respectively. In addition, the disulfide bond content of the fermented dough containing fucoidan reduced compared with that of FWD-0. The disulfide bond content in FWD-2 and FWD-3 were significantly lower than that in FWD-0 (*p* < 0.05). In addition, a higher expansion volume of the dough indicated a higher fermentation vigor of the yeast [46]. The expansion volume of the dough in FWD-2 and FWD-3 were significantly lower than those in FWD-0 (*p* < 0.05), suggesting that the fermentation vigor of FWD-0 was higher (Table 2).

The results demonstrated that the addition of fucoidan reduced the gluten protein strength of the dough. The reduced gluten protein strength might be due to the interaction between polysaccharides and gluten proteins (e.g., electrostatic interaction and hydrogen bonding), which inhibits the cross-linking of proteins to protein [47]. On the other hand, the ability of fucoidan to scavenge free radicals from the SH–SS exchange reaction reduced the amount of disulfide bonds in the dough, compromising the integrity of the gluten protein network [10]. The weakening of the integrity of the gluten protein network could lead to a decrease in the expansion volume of the dough, which in turn might affect the texture of the CSB.

### 3.3. Effect of Fucoidan Addition on Structure Properties of CSB 

#### 3.3.1. Specific Volume and Textural Property Analysis (TPA) of CSB 

TPA are essential parameters used to assess the taste of CSB and indirectly influence consumer preference for the final products [22]. The TPA of CSB with addition of fucoidan are presented in Table 3. The hardness significantly increased in CSB-1, CSB-2 and CSB-3 when compared with CSB-0 (*p* < 0.05). Likewise, a similar trend was also observed in the gumminess and chewiness of the CSB. Furthermore, the specific volume in CSB-1, CSB-2 and CSB-3 were significantly lower than those in CSB-0 (*p* < 0.05). In addition, the springiness reduced in CSB-1, CSB-2 and CSB-3 when compared with CSB-0.

The results showed that fucoidan could change the TPA and specific volume of the CSB. The polysaccharide could potentially compete for water with gluten protein, thereby preventing gluten hydration and disrupting gluten protein formation. In addition, the polysaccharide could also be introduced into the dough matrix as physical barriers, thereby inhibiting gas cell fermentation expansion [17]. Therefore, the addition of fucoidan resulted in the decrease in the specific volume of CSB. Moreover, the smaller the specific volume in the CSB, the higher the density resulted in higher hardness [48], which was consistent with our findings.

#### 3.3.2. Secondary Structure Analysis 

**Figure 2:**
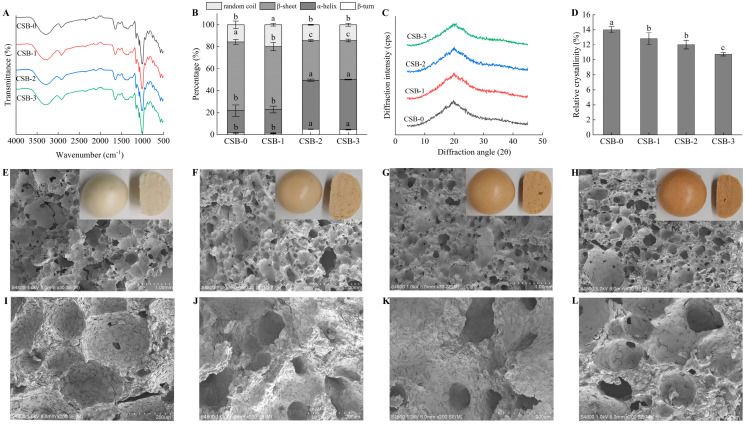
Effects of fucoidan of the structural properties of CSB. (**A**) FT-IR spectra; (**B**) Relative proportion of secondary structure; (**C**) XRD; (**D**) Relative crystallinity of starch; (**E**–**H**) SEM images of CSB samples at 30×; (**I**–**L**) SEM images of CSB samples at 200×. Different letters (a, b, c) indicate significant differences (*p* < 0.05).

The “loop and train” model of gluten protein suggested that the stable train region was primarily made up of multi-hydrogen bonded β-sheet and α-helix, while the open loop region consists of β-turn and random structures [49]. Previous research has demonstrated a correlation between dough stability with the content of β-sheet, and extensibility with the content of β-turn [50]. As shown in Figure 2A,B, the ratio of β-sheet significantly reduced in CSB-1, CSB-2 and CSB-3 when compared with CSB-0 (*p* < 0.05). As compared with CSB-0, the ratio of β-sheet in CSB-1, CSB-2 and CSB-3 decreased by 8.47%, 42.41% and 43.31%, respectively. Furthermore, the ratio of β-turn in CSB-2 and CSB-3 were significantly higher than those in CSB-0 (*p* < 0.05), and there was no significant difference between CSB-0 and CSB-1 (*p* > 0.05). It was reported that O-H stretching vibrations result in the appearance of peaks at 3600–3000 cm^−1^ due to the intramolecular or intermolecular H-bonding in gluten protein [42]. As can be seen from Figure 2A, the peak intensities of CSB-1, CSB-2 and CSB-3 decreased in 3600–3000 cm^−1^ when compared with CSB-0.

The above results indicated that the addition of a high concentration of fucoidan to CSB inhibited the formation of a gluten protein network in the CSB. It was reported that polysaccharides inhibit the development of the gluten network by competing for water molecules and breaking the hydrogen bonds between gluten and water molecules [50,51]. Therefore, fucoidan could reduce the hydrogen bonding of the gluten protein network by competing for water, weakening the gluten protein network in CSB. Thus, our study found that fucoidan could break the hydrogen bonds between gluten protein and water molecules, thereby breaking the “loop and train” model of gluten protein and inhibiting gluten protein network formation.

#### 3.3.3. Crystalline Structure Analysis

XRD analyses were used to investigate the change of starch crystallinity in the CSB during processing [19]. As shown in Figure 2C, after steaming, all of the CSB samples lost the original A-type crystalline pattern of starch, and a distinct peak emerged at around 20° (2θ). Steaming could lead to the breaking of the internal and intermolecular hydrogen bonds in the starch chain of CSB, which results in the dissociation of the helical structures of starch. Thus, the long-range crystal structure of the starches in the CSB samples was destroyed [52]. As shown in Figure 2C,D, the relative crystallinity of starch significantly reduced in CSB-1, CSB-2 and CSB-3 when compared with CSB-0 (*p* < 0.05). In addition, as compared with CSB-0, the relative crystallinity of starch in CSB-1, CSB-2 and CSB-3 decreased by 8.43%, 14.07% and 23.21%, respectively. The results of the study showed that the relative crystallinity of CSB decreased with the addition of fucoidan. Likewise, a similar trend was also observed in the Zhao et al. [53] study. On the one hand, this was the result of the polysaccharides forming a hydration layer on the surface of the starch and hindering the interaction of starch with water molecules, thereby disrupting the crystalline region of the starch [53]. On the other hand, with the addition of polysaccharides, the filling of polysaccharides between starch granules leads to a decrease in the amount of amylose leached and might impede the interaction of the amylose-amylose, thus reducing the crystallinity of starch [54]. Therefore, the relative crystallinity of starch decreased with the addition of fucoidan, which might be due to the interaction between polysaccharides and starch, thereby preventing the interaction of the amylose–amylose. 

#### 3.3.4. Microstructure Analysis

The microstructure of the CSB with the addition of fucoidan after steaming is shown in Figure 2E,L. The microstructure of CSB-1, CSB-2 and CSB-3 showed a relatively dense cross-linked structure. The size and uniformity of the gas cell in CSB-1, CSB-2 and CSB-3 were weakened when compared with CSB-0. It is well-known that the protein bodies in wheat flour, when fully hydrated, form a gluten protein network composed of glutenin and gliadin through disulfide bonds, hydrogen bonds and hydrophobic interactions. In addition, the starch was evenly distributed within the gluten protein network [55]. The scanning electron microscope results showed that the addition of fucoidan to CSB led to a denser microstructure. 

Based on the results of the above structure, it can be found that the addition of fucoidan could act on the dough system in two ways. One way might be that fucoidan formed a hydration layer on the surface of the starch granules, reducing the interaction between starch and protein molecules, thus weakening the continuity of the gluten network structure. Another way might be that fucoidan were distributed around the proteins and react with them, disrupting the integrity of the gluten protein network. These changes increased the hardness and the densification of the microstructure in the CSB.

### 3.4. Effects of Fucoidan of on the Functional Properties and Sensory of CSB

#### 3.4.1. In Vitro Starch Digestibility of CSB

**Figure 3:**
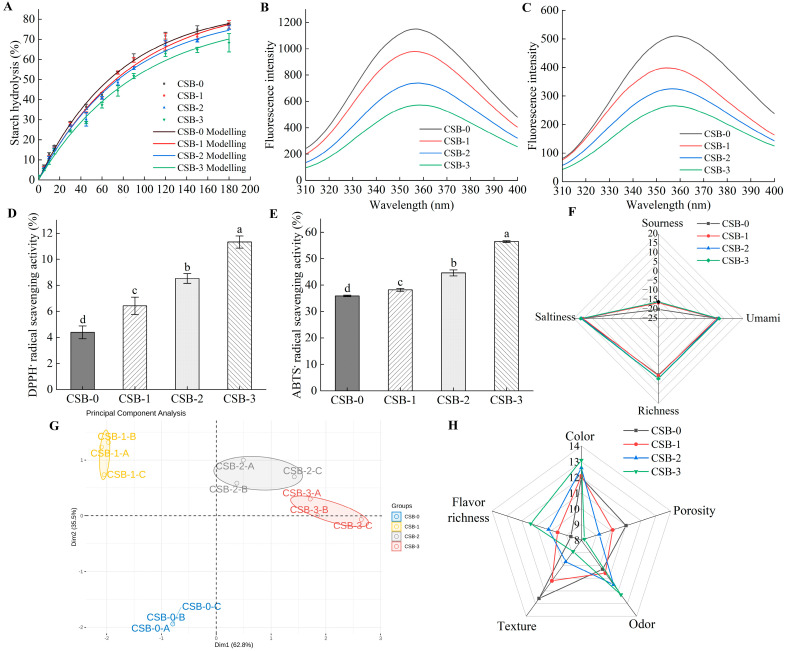
Effects of fucoidan of on the functional properties and sensory of CSB. (**A**) In vitro starch digestibility of CSB; (**B**) Fluorescence quenching of α-amylase; (**C**) Fluorescence quenching of amyloglucosidase; (**D**) DPPH· radical scavenging activity; (**E**) ABTS· radical scavenging activity; (**F**–**H**) Sensory analysis. Different letters (a, b, c, d) indicate significant differences (*p* < 0.05).

The starch digestion curve of the CSB samples in the intestinal phase is shown in Figure 3A. The data from the digestograms were fitted to the first-order rate equation to determine the first-order rate constant (k) and the equilibrium percentage of starch digestion at the end of the digestion (C_∞_). With the extension of the digestion time, the percentage of starch digestion increased gradually. Interestingly, the percentage of starch digestion in CSB-1, CSB-2 and CSB-3 showed a dose-dependent decrease compared to CSB-0. Generally, a smaller value of the first-order rate constant (k) indicates a lower percentage of starch digestion [29]. As shown in Table 4, the value of k in CSB-1, CSB-2 and CSB-3 was lower than that in CSB-0. At the same time, the pGI and HI of CSB significantly reduced in CSB-1, CSB-2 and CSB-3 when compared with CSB-0 (*p* < 0.05). It is well-known that the conformational change of proteases could be deeply understood using fluorescence spectroscopy to probe changes of the tyrosine and tryptophan residues. Thus, the binding characteristics of fucoidan with α-amylase and amyloglucosidase could be determined using fluorescence spectroscopy [56]. As shown in Figure 3B,C the fluorescence intensity of α-amylase and amyloglucosidase decreased progressively with the increase in fucoidan additions. This apparently certificated that fucoidan could interact with α-amylase or amyloglucosidase to form non-fluorescent complexes [57].

The above results showed that the addition of fucoidan in CSB might reduce the digestibility of starch. Koh et al. [12] reported that the sulfate groups of fucoidan inhibit the activity of a-amylase and amyloglucosidase by binding to the secondary site of the enzyme–substrate complex via electrostatic forces. The above results found that CSB-3 had the strongest quenching effect on α-amylase and amyloglucosidase and CSB-0 had the weakest quenching effect on α-amylase and amyloglucosidase. Fucoidan might inhibit the starch digestibility of CSB by interacting with α-amylase and amyloglucosidase. In addition, previous studies have tended to suggest that polysaccharides could wrap on the surface of starch granules to form a physical barrier or increase the viscosity in the digestive system, thereby reducing the digestibility of starch [4,58]. Thus, with the addition of fucoidan, the starch in CSB might reduce the digestibility of starch by reducing its interaction with α-amylase and amyloglucosidase. A food with a lower percentage of starch digestion would obtain a lower glycemic index value [59]. In a broader perspective, our research findings indicated that fucoidan has the potential to reduce the glycemic index. 

#### 3.4.2. Antioxidant Activity of CSB Analysis

Representative images of the antioxidant activity of each sample are shown in Figure 3D and E. The DPPH· radical scavenging activity of CSB-1, CSB-2 and CSB-3 was significantly higher than that of CSB-0 (*p* < 0.05). In addition, the ABTS· radical scavenging activity of CSB-1, CSB-2 and CSB-3 was significantly increased when compared with CSB-0 (*p* < 0.05). These findings indicate that the fucoidan-fortified CSB exhibited significant DPPH· radical scavenging activity and ABTS· radical scavenging activity. One possible explanation for the enhanced scavenging activity of polysaccharide is that the ability of polysaccharides to provide protons reduces the stable DPPH· radical to diphenylpicryl hydrazine (a type of yellow compound) and that the polysaccharide also could convert the ABTS· to a non-radical form by providing electrons [60,61]. Another plausible explanation is that the fucoidan was exposed to more sulphates during the dough fermentation process, so the antioxidant activity of the CSB was enhanced [10,15].

#### 3.4.3. Sensory Analysis

Flavor is one of the most important qualities for consumers to determine the overall acceptability and preference of CSB [62]. As shown in Figure 3F, the sourness in CSB-1, CSB-2 and CSB-3 was decreased when compared with CSB-0. Meanwhile, the richness in CSB-1, CSB-2 and CSB-3 was higher than that of CSB-0. In addition, the umami and saltiness in CSB-3 were higher than those in CSB-0. Li et al. [63] reported that the inclusion of yeast in the dough could produce succinic acid, lactic acid, acetic acid and several other metabolites such as ethanol, carbon dioxide, hydrogen peroxide, glutathione, flavor compounds and enzymes during the fermentation process. The study found that the addition of fucoidan altered the production of organic acids and flavor compounds in CSB, which would ultimately affect its taste. Figure 3G depicts the score plot of the principal component analysis of the samples, representing the discerning taste characteristics determined by the electronic tongue. The contribution rates of Dim1 and Dim2 were 62.8% and 35.5%, respectively, and the total contribution rate was 98.3%. Moreover, there was no overlap of data between different samples, representing a high degree of data dispersion [62]. This indicates that the fucoidan could effectively affect the flavor of CSB. As depicted in Figure 3H, CSB-1, CSB-2 and CSB-3 had higher flavor richness and color compared with CSB-0. Overall, fucoidan enriched the flavor of the CSB. 

## 4. Conclusions

This study aimed to investigate the potential of the addition of fucoidan (0, 1%, 3% and 5%) as functional components in CSB by characterizing the dough properties, structure properties and bioactivity in the CSB. Fucoidan could change the viscosity of unfermented dough, and high concentrations of fucoidan could remove the GS- in the dough, thereby reducing the content of disulfide bond and the expanded volume of fermented dough. Furthermore, the addition of fucoidan inhibited the aggregation of gluten proteins, thus increasing the chewiness of the CSB. This was due to the fact that fucoidan forms a physical barrier on the surface of the starch particles and hinder the reaction between protein-to-protein, thus breaking the “loop and train” mode of gluten. More importantly, the interaction of fucoidan with the dough system resulted in lower starch digestibility and higher antioxidant activity of the CSB. This study could provide a theoretical basis for the diversified application of fucoidan in wheat-based foods. Further research is required to explore the mechanism of fucoidan in reducing blood sugar and improving the antioxidant activity through in vivo and in vitro experiments.

## Figures and Tables

**Table 1:** References used to train the panelists.

Descriptors	Sensory Score
Color	Low vividness (0–4) More vividness (5–10) Strong vividness (11–15)
Porosity of the crumb (pore size)	Small (0–4) Comparatively small (5–10) Big (11–15)
Odor	CSB with weak odor (0–4) CSB with Comparatively weak odor (5–10) CSB with strong odor (11–15)
Texture (by feeling it with hands) (a) Softness-force required to compress sample between fingers; (b) Springiness-swiftness of returning to the initial shape after moderate pressure applied to the center of the CSB; (c) Crookedness-presence of cracks on the surface of the samples	Poor texture (0–4) Good texture (5–10) Better texture (11–15)
Flavor richness (Eating a variety of flavor in a CSB)	Weak (0–4) Weaker (5–10) Strong (11–15)

**Table 2:** Effect of fucoidan addition on the SH, SS and expansion volume of fermented dough.

Sample	SH (μmol/g)	SS (μmol/g)	Expansion Volume (mL)
FWD-0	4.14 ± 0.06 ^d^	59.80 ± 1.09 ^a^	48.67 ± 4.16 ^a^
FWD-1	4.26 ± 0.02 ^c^	59.28 ± 0.86 ^a^	46.00 ± 2.00 ^ab^
FWD-2	4.50 ± 0.04 ^b^	51.23 ± 2.93 ^b^	42.67 ± 2.31 ^b^
FWD-3	4.78 ± 0.06 ^a^	50.99 ± 3.22 ^b^	37.33 ± 1.15 ^c^

FWD-0, fermented wheat dough without fucoidan; FWD-1, fermented wheat dough with 1% fucoidan; FWD-2, fermented wheat dough with 3% fucoidan; FWD-3, fermented wheat dough with 5% fucoidan. Different letters indicate significant differences (*p* < 0.05).

**Table 3:** Effect of different additions of fucoidan on the TPA and specific volume of CSB.

Sample	Hardness (g)	Springiness	Gumminess	Chewiness	Specific Volume (cm^3^/g)
CSB-0	252.67 ± 11.12 ^c^	4.76 ± 0.15 ^a^	219.17 ± 14.31 ^c^	10.22 ± 0.69 ^c^	2.48 ± 0.26 ^a^
CSB-1	439.00 ± 55.00 ^b^	3.86 ± 0.10 ^c^	395.00 ± 47.18 ^b^	15.13 ± 1.88 ^b^	1.95 ± 0.07 ^b^
CSB-2	491.83 ± 69.03 ^ab^	4.08 ± 0.40 ^bc^	418.00 ± 33.00 ^b^	17.22 ± 1.53 ^b^	2.12 ± 0.12 ^b^
CSB-3	549.00 ± 24.07 ^a^	4.31 ± 0.12 ^b^	487.07 ± 19.31 ^a^	20.59 ± 0.68 ^a^	2.01 ± 0.10 ^b^

CSB with 0, 1%, 3%, 5% of fucoidan was named as CSB-0, CSB-1, CSB-2 and CSB-3, respectively. Different letters indicate significant differences (*p* < 0.05).

**Table 4:** Modeling parameters of in vitro starch digestibility curve.

	CSB-0	CSB-1	CSB-2	CSB-3
C_∞_ (%)	88.70 ± 0.92 ^a^	87.34 ± 2.31 ^a^	88.63± 0.91 ^a^	81.47 ± 5.31 ^b^
K (10^−2^ cm^−1^)	1.24 ± 0.04 ^a^	1.19 ± 0.06 ^ab^	1.08 ± 0.04 ^b^	1.08 ± 0.10 ^b^
R^2^	0.9997	0.9992	0.9995	0.9993
HI/%	99.49 ± 0.52 ^a^	96.17 ± 0.46 ^b^	92.77 ± 0.85 ^c^	85.12 ± 1.10 ^d^
pGI	94.33 ± 0.29 ^a^	92.51 ± 0.25 ^b^	90.64 ± 0.47 ^c^	86.44 ± 0.60 ^d^

Different letters indicate significant differences (*p* < 0.05).

## Data Availability

The original contributions presented in the study are included in the article, further inquiries can be directed to the corresponding authors.
